# Crystal structure of *cyclo*-tris­(μ-3,4,5,6-tetra­fluoro-*o*-phenyl­ene-κ^2^ *C* ^1^:*C* ^2^)trimercury–tetra­cyano­ethyl­ene (1/1)

**DOI:** 10.1107/S2056989015019350

**Published:** 2015-10-24

**Authors:** Raúl Castañeda, Tatiana V. Timofeeva, Victor N. Khrustalev

**Affiliations:** a Department of Chemistry & Biology, New Mexico Highlands University, 803 University Ave., Las Vegas, NM 87701, USA; b Inorganic Chemistry Department, Peoples’ Friendship University of Russia, 6 Miklukho-Maklay St, Moscow, 117198, Russian Federation

**Keywords:** crystal structure, trimeric perfluoro-*o*-phenyl­ene mercury, tetra­cyano­ethyl­ene, complexation, X-ray diffraction, TGA

## Abstract

The title compound, [Hg_3_(C_6_F_4_)_3_]·C_6_N_4_, contains one mol­ecule of tetra­cyano­ethyl­ene **B** per one mol­ecule of mercury macrocycle **A**, *i.e.*, **A•B**, and crystallizes in the monoclinic space group *C*2/*c*. Macrocycle **A** and mol­ecule **B** both occupy special positions on a twofold rotation axis and the inversion centre, respectively. The supra­molecular unit [**A•B**] is built by the simultaneous coordination of one of the nitrile N atoms of **B** to the three mercury atoms of the macrocycle **A**. The Hg⋯N distances range from 2.990 (4) to 3.030 (4) Å and are very close to those observed in the related adducts of the macrocycle **A** with other nitrile derivatives. The mol­ecule of **B** is almost perpendicular to the mean plane of the macrocycle **A** at the dihedral angle of 88.20 (5)°. The donor–acceptor Hg⋯N inter­actions do not affect the C N bond lengths [1.136 (6) and 1.140 (6) Å]. The *trans* nitrile group of **B** coordinates to another macrocycle **A**, forming an infinite mixed-stack [**A•B**]_∞_ architecture toward [101]. The remaining N atoms of two nitrile groups of **B** are not engaged in any donor–acceptor inter­actions. In the crystal, the mixed stacks are held together by inter­molecular C—F⋯C N secondary inter­actions [2.846 (5)–2.925 (5) Å].

## Abstract 2

The crystal structure and thermal properties of a mixed-stack donor–acceptor complex of trimeric perfluoro-*o*-phenyl­ene mercury with tetra­cyano­ethyl­ene in an 1:1 ratio were studied by X-ray diffraction and TGA methods.

## Chemical context   

Trimeric perfluoro-*o*-phenyl­ene mercury (**A**) is a versatile Lewis acid that is applied for complexation with different substrates, in particular, for the obtaining of charge-transfer complexes based on donor–acceptor inter­molecular inter­actions (Hasegawa *et al.*, 2004 ▸). Importantly, some physical properties of the guest substrates can change upon complexation. For example, unusual optical properties of the organic mol­ecules in supra­molecular complexes with macrocycle **A** have previously been observed (Haneline *et al.*, 2002 ▸; Elbjeirami *et al.*, 2007 ▸; Filatov *et al.*, 2009 ▸, 2011 ▸). Moreover, using complexation with **A**, the stabilization of different organic (di­phenyl­polyynes; Taylor & Gabbaï, 2006 ▸; Taylor *et al.*, 2008 ▸) and metal-organic (nickelocene; Haneline & Gabbaï, 2004*a* ▸) mol­ecules was achieved under ambient conditions. In this paper, a complex of **A** with tetra­cyano­ethyl­ene (**B**) – an unstable dienophilic (σ-electron donor and π-electron acceptor) compound – [Hg_3_(C_6_F_4_)_3_]·C_6_N_4_, (**I**), was prepared and studied by X-ray diffraction analysis to get a deeper understanding of the complexation process.

## Structural commentary   

**Figure 1:**
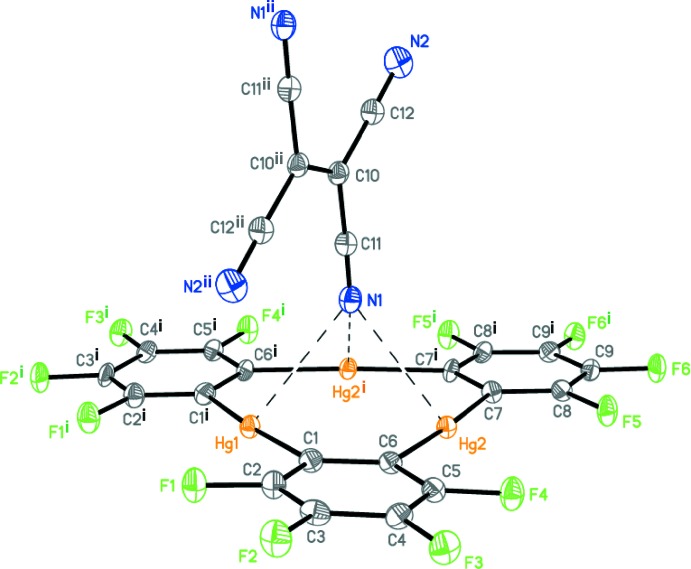
The supra­molecular unit of complex (**I**) (**A•B**). Displacement ellipsoids are drawn at the 50% probability level. Dashed lines indicate the inter­molecular secondary Hg⋯N inter­actions. [Symmetry codes: (i) 1 − *x*, *y*, 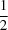 − *z*; (ii) 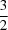 − *x*, 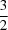 − *y*, 1 − *z*.]

Complex (**I**) contains one mol­ecule of tetra­cyano­ethyl­ene **B** per one mol­ecule of the mercury macrocycle **A**, *i.e.*, C_18_F_12_Hg_3_·C_6_N_4_ (**A•B**), and crystallizes in the monoclinic space group *C*2/*c*. Both macrocycle **A** and the mol­ecule of **B** occupy special positions on a twofold rotation axis and inversion centre, respectively. The supra­molecular unit of (**I**) is built by the simultaneous coordination of the nitrile N1 nitro­gen atom of **B** to the three mercury atoms of the macrocycle **A** (Fig. 1 ▸). The Hg⋯N distances range from 2.990 (4) to 3.030 (4) Å and are very close to those observed in related adducts of macrocycle **A** with other nitrile derivatives: aceto­nitrile [2.93 (1)–2.99 (1) Å], acrylo­nitrile [2.87 (1)–2.96 (1) Å] and benzo­nitrile [2.97 (1)–3.13 (1) Å] (Tikhonova *et al.*, 2000 ▸) and 7,7,8,8-tetra­cyano­quinodi­methane (**II**) [3.102 (11)–3.134 (11) Å] (Haneline & Gabbaï, 2004*b* ▸). Thus, the N1 nitro­gen atom is essentially equidistant to the three Lewis acidic sites of the macrocycle **A**. The mol­ecule of **B** is almost perpendicular to the mean plane of macrocycle **A**, making a dihedral angle of 88.20 (5)°. It is very important to point out that the donor–acceptor Hg⋯N inter­actions do not affect the C N bond lengths [1.136 (6) and 1.140 (6) Å].
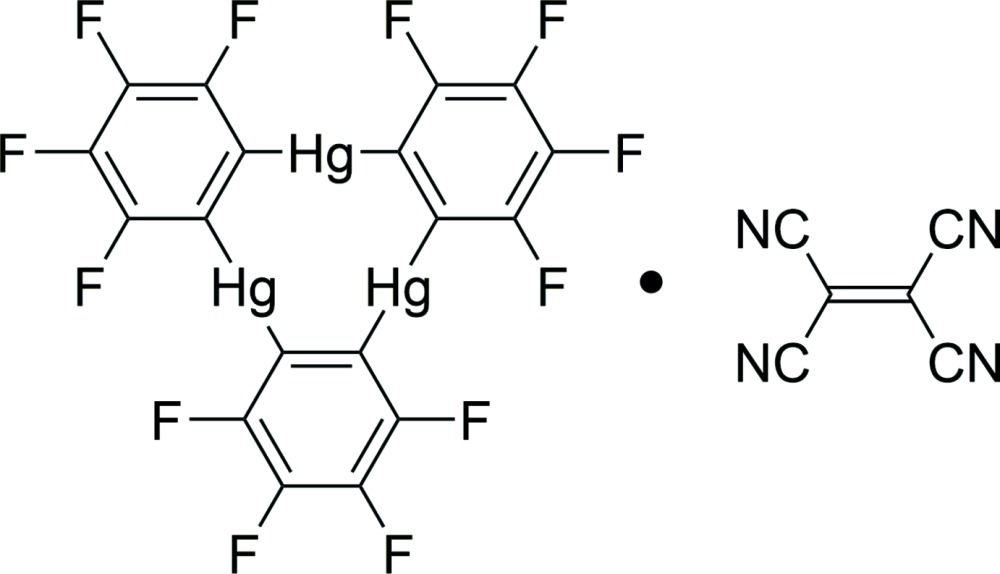


**Figure 2:**
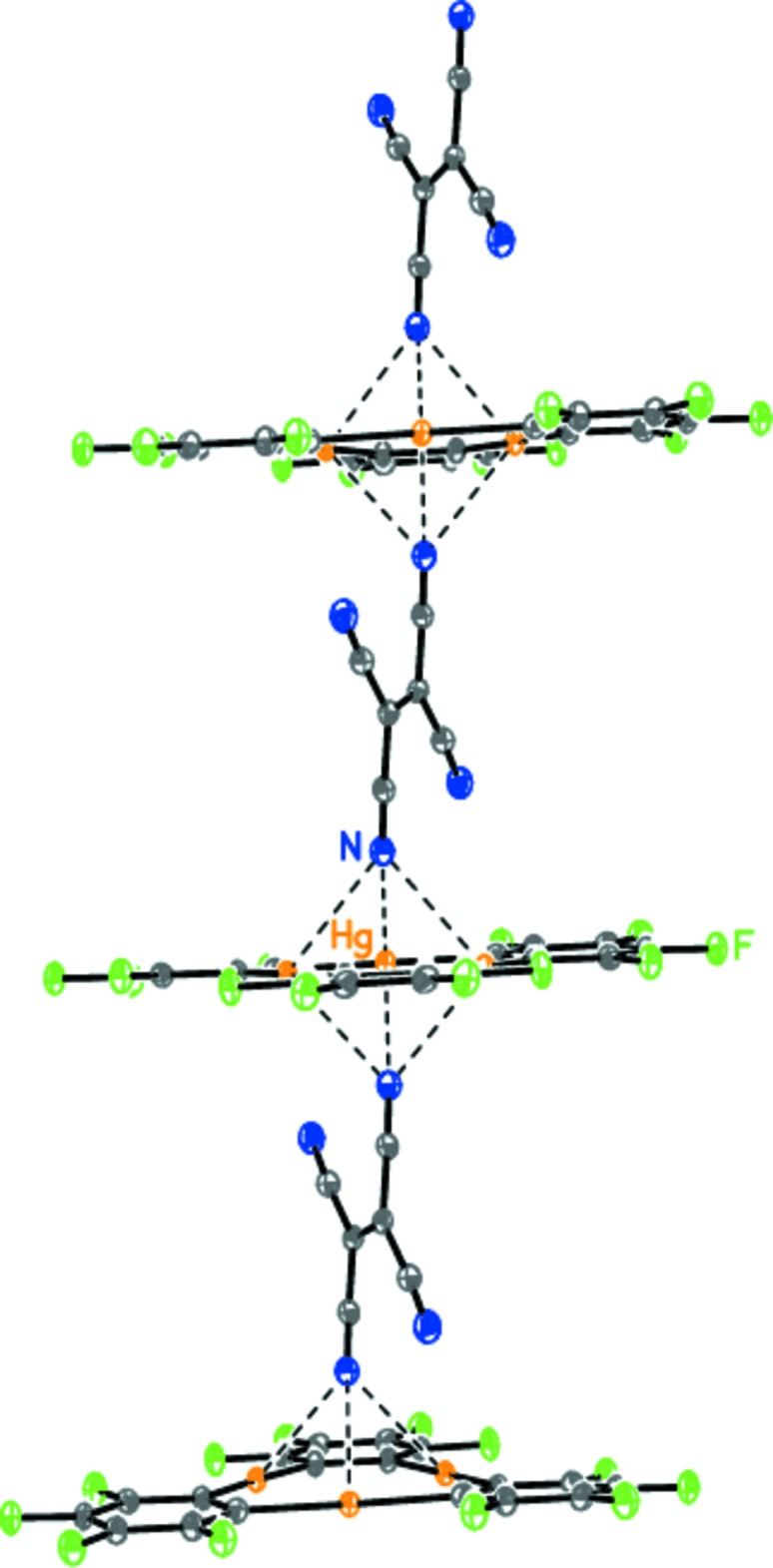
The infinite mixed-stack [**A•B**]_∞_ architecture of (**I**). Dashed lines indicate the inter­molecular secondary Hg⋯N inter­actions.

Taking into account the intrinsic *C_i_* symmetry of **B**, the *trans* nitrile group of this mol­ecule coordinates to another macrocycle **A**, forming an infinite mixed-stack [**A•B**]_∞_ architecture (Fig. 2 ▸). The remaining nitro­gen atoms of the two nitrile groups of **B** are not engaged in any donor–acceptor inter­actions.

## Supra­molecular features   

**Figure 3:**
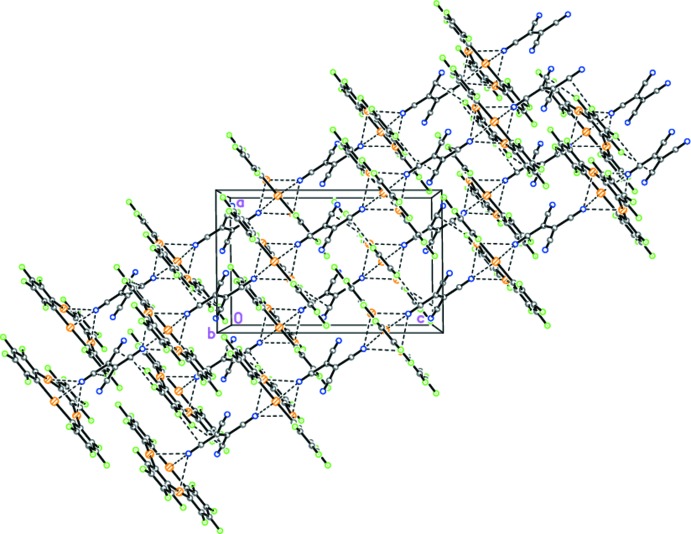
Crystal packing of complex (**I**) along the *b* axis, showing the infinite mixed stacks toward [101]. Dashed lines indicate the inter­molecular secondary Hg⋯N and F⋯C inter­actions.

In the crystal, the mixed stacks toward [101] are held together by inter­molecular C—F⋯C N secondary inter­actions [F2⋯C11^iii^ 2.864 (5), F5⋯C12^iv^ 2.846 (5) and F6⋯C11^v^ 2.925 (5) Å; symmetry codes: (iii) −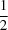 + *x*, 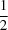 + *y*, *z*; (iv) 1 − *x*, 1 − *y*, 1 − *z*; (v) −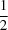 + *x*, −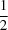 + *y*, *z*] (Fig. 3 ▸).

## Comparison with compound (**II**)   

**Figure 4:**
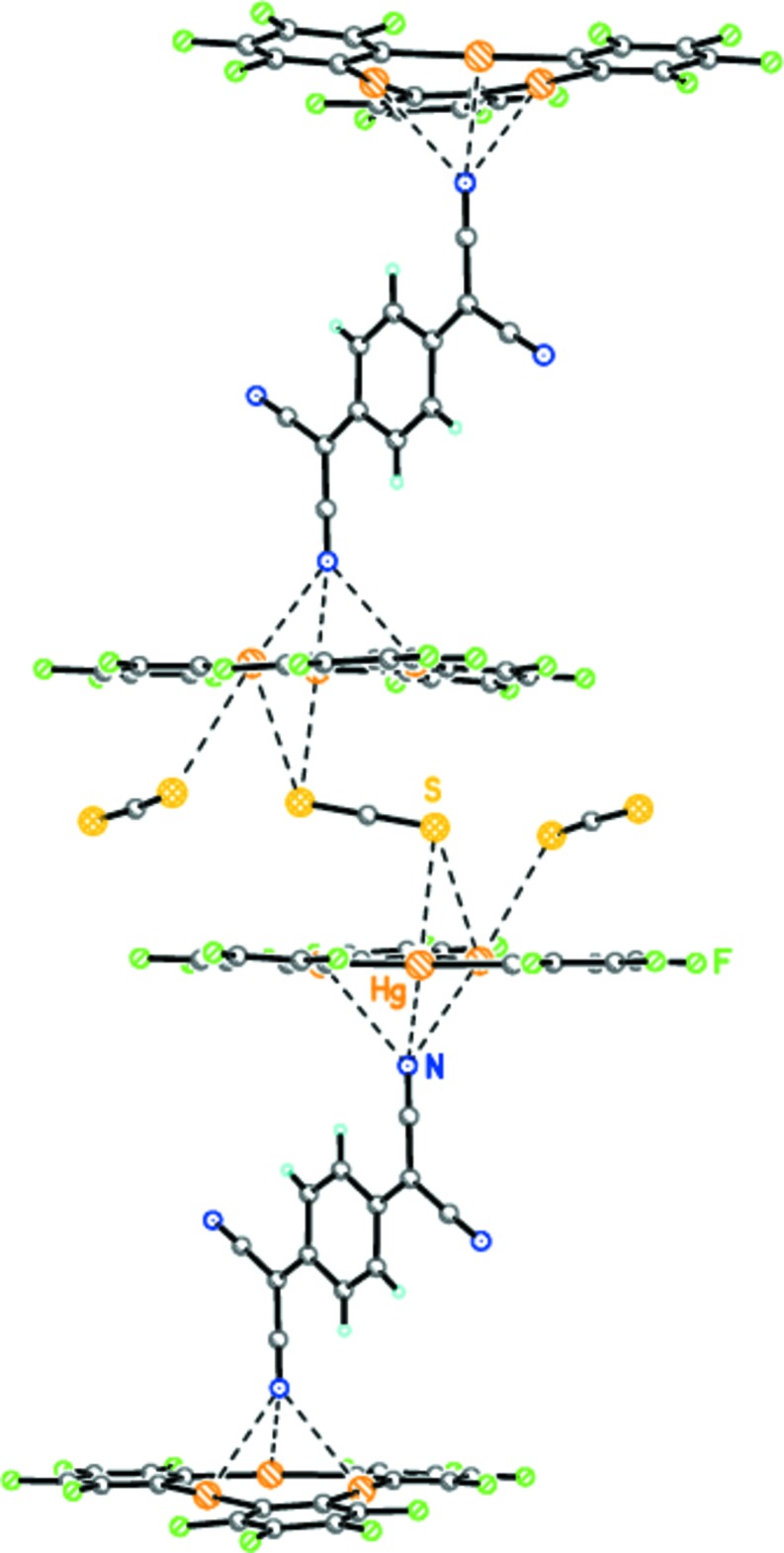
The supra­molecular structure of complex (**II**) ([**A•C•A•1.5D]**_∞_). Dashed lines indicate the inter­molecular secondary Hg⋯N inter­actions.

It is inter­esting to note that the crystal structures of (**I**) and (**II**) are very similar. In both complexes, the guest mol­ecules of tetra­cyano­ethyl­ene **B** and tetra­cyano­quinodi­methane **C** are arranged perpendicularly to macrocycle **A**, with the same coordination mode of the *trans* nitrile groups to the three mercury atoms (Fig. 4 ▸). However, the supra­molecular unit in (**I**) is **A•B** (a 1:1 ratio), whereas that in (**II**) is **A•C•A** (a 2:1 ratio) (Fig. 4 ▸). Beside the mol­ecules of **C**, complex (**II**) includes the additional solvate CS_2_ (**D**) mol­ecules. The mol­ecules of **D** participate in the construction of the supra­molecular architecture of (**II**), resulting in infinite mixed stacks [**A•C•A•1.5D]**_∞_ (Fig. 4 ▸). Remarkably, the total number of donor–acceptor inter­molecular inter­actions within the infinite mixed stacks of (**I**) and (**II**) is equal ([12 Hg⋯N]_∞_ and [6 Hg⋯N + 6 Hg⋯S]_∞_, respectively).

## TGA analysis   

**Figure 5:**
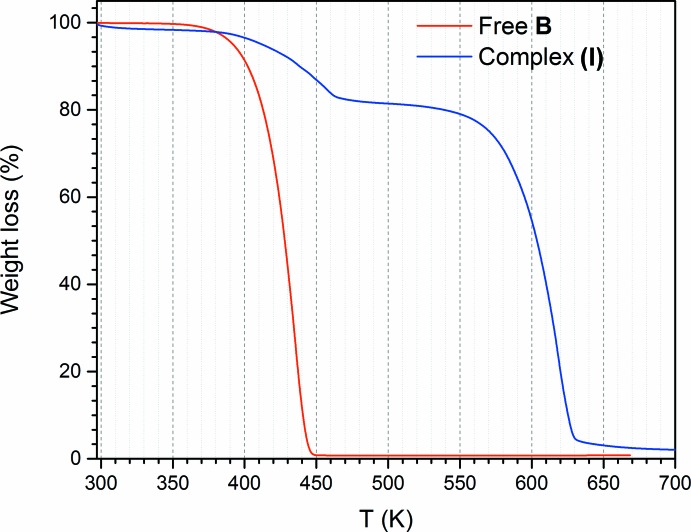
TGA diagram of free **B** (in red) and complex (**I**) (in blue).

Despite complexes (**I**) and (**II**) being structural analogs, they are substanti­ally different in their chemical stability. The crystalline complex (**II**) decomposes over a few days, while complex (**I**) is stable in the solid state for several months under ambient conditions. As free **B** decomposes rapidly upon reaction with moisture to produce toxic hydrogen cyanide, the high chemical stability of complex (**I**) is surprising. Moreover, the thermal stability of complex (**I**) has been studied by thermogravimetric analysis (TGA) which revealed that, upon complexation, tetra­cyano­ethyl­ene is stable to higher temperatures (Fig. 5 ▸). So, the free compound **B** starts to decompose at 363 K, but, being incorporated into the supra­molecular complex (**I**), **B** is stable up to 393 K. Complex (**I**) decomposes in two different steps. The first step of a 18.3% weight loss is attributed to molecule **B** because the much lower decomposition temperature of this molecule compared to macrocycle **A**. Consequently, the second weight loss of 81.7% is attributed to decomposition of macrocycle **A**. The complete decomposition of the free **B** is complete at 445 K; however, its final decomposition temperature is equal to 467 K within the supra­molecular complex (**I**). Final decomposition of complex (**I**) occurs at 573 K, and is likely due decomposition of macrocycle **A**.

It is known that tetra­cyano­ethyl­ene is used not only as a component of charge-transfer complexes for organic electronics, but also in the preparation of organic magnets (Kao *et al.*, 2012 ▸). Consequently, the increase of its thermal stability attracts special attention in the manufacturing of organic materials. The complexation method described here could help to solve this problem.

## Synthesis and crystallization   

Trimeric per­fluoro-*o*-phenyl­ene mercury was synthesized according to the procedure described previously (Sartori & Golloch, 1968 ▸), and purified by recrystallization in di­chloro­methane (Filatov *et al.*, 2009 ▸). Tetra­cyano­ethyl­ene was acquired from TCI America. All solvents were HPLC grade and used without any further purification. Thermogravimetric analysis was performed with a Hitachi STA7200 SII NanoTechnology instrument (an aluminum crucible (45 mL) was used; heating rate was 10 K min^−1^).

Stoichiometric amounts of trimeric per­fluoro-*o*-phenyl­ene mercury (63.8 mg, 59.6 mmol) and tetra­cyano­ethyl­ene (7.7 mg, 59.6 mmol) were dissolved in di­chloro­methane in separate tubes using ultrasonication. The contents of the tubes were mixed carefully, and then left for slow evaporation of the solvents. Complex (**I)** was obtained as yellow prismatic crystals, m*.*p. = 499–500 K.

## Refinement   

Crystal data, data collection and structure refinement details are summarized in Table 1 ▸. There is a high positive residual density of 1.19–1.78 e Å^−3^ near the Hg1 and Hg2 atoms due to considerable absorption effects which could not be completely corrected.

## Supplementary Material

Crystal structure: contains datablock(s) global, I. DOI: 10.1107/S2056989015019350/ru2064sup1.cif

Structure factors: contains datablock(s) I. DOI: 10.1107/S2056989015019350/ru2064Isup2.hkl

CCDC reference: 1430883

Additional supporting information:  crystallographic information; 3D view; checkCIF report

## Figures and Tables

**Table 1:** Experimental details

Crystal data	
Chemical formula	[Hg_3_(C_6_F_4_)_3_]C_6_N_4_
*M*_r_	1174.05
Crystal system, space group	Monoclinic, *C*2/*c*
Temperature (K)	100
*a*, *b*, *c* ()	10.5658(11), 13.8297(15), 16.7166(18)
()	90.575(1)
*V* (^3^)	2442.5(5)
*Z*	4
Radiation type	Mo *K*
(mm^1^)	18.93
Crystal size (mm)	0.15 0.15 0.10

Data collection	
Diffractometer	Bruker APEXII CCD
Absorption correction	Multi-scan (*SADABS*; Bruker, 2003 ▸)
*T*_min_, *T*_max_	0.150, 0.250
No. of measured, independent and observed [*I* > 2(*I*)] reflections	13813, 3560, 3404
*R*_int_	0.036
(sin /)_max_ (^1^)	0.703

Refinement	
*R*[*F* ^2^ > 2(*F* ^2^)], *wR*(*F* ^2^), *S*	0.023, 0.055, 1.06
No. of reflections	3560
No. of parameters	195
_max_, _min_ (e ^3^)	1.78, 1.70

Computer programs: *APEX2* (Bruker, 2005 ▸), *SAINT* (Bruker, 2001 ▸), *SHELXTL* (Sheldrick, 2008 ▸) and *SHELXL2014* (Sheldrick, 2015 ▸).

## supplementary crystallographic information

### Crystal data

**Table:**

[Hg_3_(C_6_F_4_)_3_]·C_6_N_4_	*F*(000) = 2080
*M**_r_* = 1174.05	*D*_x_ = 3.193 Mg m^−^^3^
Monoclinic, *C*2/*c*	Mo *K*α radiation, λ = 0.71073 Å
*a* = 10.5658 (11) Å	Cell parameters from 9624 reflections
*b* = 13.8297 (15) Å	θ = 4.4–32.7°
*c* = 16.7166 (18) Å	µ = 18.93 mm^−^^1^
β = 90.575 (1)°	*T* = 100 K
*V* = 2442.5 (5) Å^3^	Prism, light-yellow
*Z* = 4	0.15 × 0.15 × 0.10 mm

### Data collection

**Table:**

Bruker APEXII CCD diffractometer	3404 reflections with *I* > 2σ(*I*)
Radiation source: fine-focus sealed tube	*R*_int_ = 0.036
φ and ω scans	θ_max_ = 30.0°, θ_min_ = 4.4°
Absorption correction: multi-scan (*SADABS*; Bruker, 2003)	*h* = −14→14
*T*_min_ = 0.150, *T*_max_ = 0.250	*k* = −19→19
13813 measured reflections	*l* = −23→23
3560 independent reflections	

### Refinement

**Table:**

Refinement on *F*^2^	0 restraints
Least-squares matrix: full	Primary atom site location: difference Fourier map
*R*[*F*^2^ > 2σ(*F*^2^)] = 0.023	Secondary atom site location: difference Fourier map
*wR*(*F*^2^) = 0.055	*w* = 1/[σ^2^(*F*_o_^2^) + (0.0191*P*)^2^ + 19.1*P*] where *P* = (*F*_o_^2^ + 2*F*_c_^2^)/3
*S* = 1.06	(Δ/σ)_max_ = 0.002
3560 reflections	Δρ_max_ = 1.78 e Å^−^^3^
195 parameters	Δρ_min_ = −1.70 e Å^−^^3^

### Special details

**Table:**

Geometry. All e.s.d.'s (except the e.s.d. in the dihedral angle between two l.s. planes) are estimated using the full covariance matrix. The cell e.s.d.'s are taken into account individually in the estimation of e.s.d.'s in distances, angles and torsion angles; correlations between e.s.d.'s in cell parameters are only used when they are defined by crystal symmetry. An approximate (isotropic) treatment of cell e.s.d.'s is used for estimating e.s.d.'s involving l.s. planes.

### Fractional atomic coordinates and isotropic or equivalent isotropic displacement parameters (Å^2^)

**Table:**

	*x*	*y*	*z*	*U*_iso_*/*U*_eq_	
Hg1	0.5000	0.83281 (2)	0.2500	0.01573 (6)	
Hg2	0.36631 (2)	0.60249 (2)	0.31557 (2)	0.01493 (5)	
F1	0.3423 (3)	0.9966 (2)	0.33993 (17)	0.0259 (6)	
F2	0.1469 (3)	0.98904 (19)	0.44275 (17)	0.0267 (6)	
F3	0.0409 (3)	0.8182 (2)	0.48130 (17)	0.0253 (5)	
F4	0.1388 (3)	0.65100 (19)	0.42523 (17)	0.0247 (5)	
F5	0.3016 (3)	0.38265 (19)	0.35217 (16)	0.0240 (5)	
F6	0.4034 (3)	0.21483 (18)	0.30301 (17)	0.0238 (5)	
C1	0.3490 (4)	0.8248 (3)	0.3293 (3)	0.0186 (7)	
C2	0.2968 (4)	0.9087 (3)	0.3601 (3)	0.0198 (8)	
C3	0.1952 (4)	0.9069 (3)	0.4121 (3)	0.0199 (8)	
C4	0.1415 (4)	0.8193 (3)	0.4332 (3)	0.0191 (8)	
C5	0.1927 (4)	0.7350 (3)	0.4030 (2)	0.0177 (7)	
C6	0.2958 (4)	0.7350 (3)	0.3517 (2)	0.0153 (7)	
C7	0.4474 (4)	0.4743 (3)	0.2765 (2)	0.0161 (7)	
C8	0.4008 (4)	0.3863 (3)	0.3008 (2)	0.0173 (7)	
C9	0.4501 (4)	0.2987 (3)	0.2762 (3)	0.0189 (8)	
N1	0.6254 (4)	0.6785 (3)	0.3521 (2)	0.0213 (7)	
N2	0.9176 (4)	0.5836 (3)	0.5355 (3)	0.0289 (8)	
C10	0.7588 (4)	0.7081 (3)	0.4799 (2)	0.0165 (7)	
C11	0.6861 (4)	0.6895 (3)	0.4081 (2)	0.0174 (7)	
C12	0.8478 (4)	0.6369 (3)	0.5080 (2)	0.0198 (7)	

### Atomic displacement parameters (Å^2^)

**Table:**

	*U*^11^	*U*^22^	*U*^33^	*U*^12^	*U*^13^	*U*^23^
Hg1	0.01452 (9)	0.01481 (10)	0.01793 (10)	0.000	0.00398 (7)	0.000
Hg2	0.01430 (7)	0.01349 (8)	0.01709 (8)	0.00109 (4)	0.00400 (5)	0.00000 (5)
F1	0.0273 (13)	0.0178 (12)	0.0327 (14)	−0.0010 (10)	0.0092 (11)	0.0003 (10)
F2	0.0296 (14)	0.0186 (13)	0.0321 (14)	0.0050 (10)	0.0088 (11)	−0.0055 (10)
F3	0.0209 (12)	0.0275 (14)	0.0277 (13)	0.0033 (10)	0.0111 (10)	−0.0001 (11)
F4	0.0243 (13)	0.0192 (12)	0.0308 (14)	−0.0003 (10)	0.0110 (10)	0.0012 (10)
F5	0.0237 (13)	0.0204 (13)	0.0281 (13)	−0.0003 (10)	0.0139 (10)	0.0032 (10)
F6	0.0251 (13)	0.0132 (12)	0.0333 (14)	−0.0026 (9)	0.0073 (11)	0.0029 (10)
C1	0.0174 (18)	0.0188 (19)	0.0195 (18)	0.0010 (14)	0.0016 (14)	0.0010 (14)
C2	0.0200 (19)	0.0171 (19)	0.0225 (19)	0.0003 (14)	0.0045 (15)	−0.0001 (15)
C3	0.0190 (18)	0.0184 (19)	0.0223 (19)	0.0091 (14)	0.0018 (15)	−0.0041 (15)
C4	0.0142 (17)	0.023 (2)	0.0206 (18)	0.0024 (14)	0.0054 (14)	−0.0003 (15)
C5	0.0181 (18)	0.0180 (18)	0.0171 (17)	0.0014 (14)	0.0023 (14)	0.0010 (14)
C6	0.0142 (16)	0.0158 (18)	0.0161 (17)	0.0034 (13)	0.0031 (13)	−0.0007 (13)
C7	0.0148 (17)	0.0149 (17)	0.0186 (17)	0.0038 (13)	0.0053 (14)	0.0002 (14)
C8	0.0159 (17)	0.0188 (19)	0.0173 (17)	0.0026 (14)	0.0035 (14)	0.0019 (14)
C9	0.0177 (18)	0.0161 (18)	0.0228 (19)	−0.0020 (14)	0.0001 (15)	0.0018 (15)
N1	0.0216 (17)	0.0193 (17)	0.0231 (17)	−0.0011 (13)	0.0044 (13)	0.0001 (13)
N2	0.028 (2)	0.028 (2)	0.031 (2)	0.0051 (16)	0.0072 (16)	0.0016 (16)
C10	0.0143 (16)	0.0169 (18)	0.0184 (17)	−0.0012 (13)	0.0030 (13)	0.0010 (14)
C11	0.0182 (18)	0.0166 (18)	0.0174 (17)	−0.0001 (14)	0.0036 (14)	−0.0011 (14)
C12	0.0196 (18)	0.0195 (19)	0.0205 (18)	0.0001 (15)	0.0030 (14)	−0.0006 (15)

### Geometric parameters (Å, º)

**Table:**

Hg1—C1	2.087 (4)	C2—C3	1.389 (6)
Hg1—N1	3.030 (4)	C3—C4	1.385 (6)
Hg2—C6	2.071 (4)	C4—C5	1.382 (6)
Hg2—C7	2.077 (4)	C5—C6	1.394 (5)
Hg2—N1	2.990 (4)	C7—C8	1.375 (6)
F1—C2	1.351 (5)	C7—C7^i^	1.429 (7)
F2—C3	1.348 (5)	C8—C9	1.384 (6)
F3—C4	1.340 (4)	C9—C9^i^	1.377 (8)
F4—C5	1.347 (5)	N1—C11	1.140 (6)
F5—C8	1.363 (5)	N2—C12	1.136 (6)
F6—C9	1.339 (5)	C10—C10^ii^	1.355 (8)
C1—C2	1.387 (6)	C10—C12	1.437 (6)
C1—C6	1.415 (6)	C10—C11	1.441 (6)
			
C1—Hg1—C1^i^	173.9 (2)	C1—C6—Hg2	123.7 (3)
C6—Hg2—C7	176.24 (16)	C8—C7—C7^i^	117.8 (2)
C2—C1—C6	118.4 (4)	C8—C7—Hg2	120.8 (3)
C2—C1—Hg1	120.0 (3)	C7^i^—C7—Hg2	121.39 (11)
C6—C1—Hg1	121.6 (3)	F5—C8—C7	119.9 (3)
F1—C2—C1	121.1 (4)	F5—C8—C9	116.7 (4)
F1—C2—C3	116.8 (4)	C7—C8—C9	123.4 (4)
C1—C2—C3	122.1 (4)	F6—C9—C9^i^	120.0 (2)
F2—C3—C4	118.9 (4)	F6—C9—C8	121.2 (4)
F2—C3—C2	121.4 (4)	C9^i^—C9—C8	118.8 (2)
C4—C3—C2	119.7 (4)	C11—N1—Hg2	136.2 (3)
F3—C4—C5	121.7 (4)	C11—N1—Hg1	127.4 (3)
F3—C4—C3	119.5 (4)	Hg2—N1—Hg1	74.82 (9)
C5—C4—C3	118.9 (4)	C10^ii^—C10—C12	121.1 (5)
F4—C5—C4	117.3 (4)	C10^ii^—C10—C11	119.4 (5)
F4—C5—C6	120.3 (4)	C12—C10—C11	119.5 (4)
C4—C5—C6	122.4 (4)	N1—C11—C10	176.9 (5)
C5—C6—C1	118.6 (4)	N2—C12—C10	175.2 (5)
C5—C6—Hg2	117.7 (3)		
			
C6—C1—C2—F1	−179.0 (4)	F4—C5—C6—C1	179.4 (4)
Hg1—C1—C2—F1	−0.2 (6)	C4—C5—C6—C1	−0.6 (6)
C6—C1—C2—C3	0.3 (6)	F4—C5—C6—Hg2	−2.5 (5)
Hg1—C1—C2—C3	179.1 (3)	C4—C5—C6—Hg2	177.5 (3)
F1—C2—C3—F2	−1.9 (6)	C2—C1—C6—C5	0.7 (6)
C1—C2—C3—F2	178.8 (4)	Hg1—C1—C6—C5	−178.1 (3)
F1—C2—C3—C4	177.9 (4)	C2—C1—C6—Hg2	−177.2 (3)
C1—C2—C3—C4	−1.4 (7)	Hg1—C1—C6—Hg2	4.0 (5)
F2—C3—C4—F3	2.0 (6)	C7^i^—C7—C8—F5	179.2 (4)
C2—C3—C4—F3	−177.8 (4)	Hg2—C7—C8—F5	−1.1 (5)
F2—C3—C4—C5	−178.7 (4)	C7^i^—C7—C8—C9	−0.4 (7)
C2—C3—C4—C5	1.5 (6)	Hg2—C7—C8—C9	179.3 (3)
F3—C4—C5—F4	−1.2 (6)	F5—C8—C9—F6	−1.2 (6)
C3—C4—C5—F4	179.5 (4)	C7—C8—C9—F6	178.4 (4)
F3—C4—C5—C6	178.8 (4)	F5—C8—C9—C9^i^	179.5 (5)
C3—C4—C5—C6	−0.5 (6)	C7—C8—C9—C9^i^	−0.8 (8)

Symmetry codes: (i) −*x*+1, *y*, −*z*+1/2; (ii) −*x*+3/2, −*y*+3/2, −*z*+1.
